# Functions of the RNA Editing Enzyme ADAR1 and Their Relevance to Human Diseases

**DOI:** 10.3390/genes7120129

**Published:** 2016-12-17

**Authors:** Chunzi Song, Masayuki Sakurai, Yusuke Shiromoto, Kazuko Nishikura

**Affiliations:** The Wistar Institute, 3601 Spruce Street, Philadelphia, PA 19104, USA; msakurai@wistar.org (M.S.); yshiromoto@wistar.org (Y.S.); kazuko@wistar.org (K.N.)

**Keywords:** ADAR1, RNA editing, human diseases, innate immunity

## Abstract

Adenosine deaminases acting on RNA (ADARs) convert adenosine to inosine in double-stranded RNA (dsRNA). Among the three types of mammalian ADARs, ADAR1 has long been recognized as an essential enzyme for normal development. The interferon-inducible ADAR1p150 is involved in immune responses to both exogenous and endogenous triggers, whereas the functions of the constitutively expressed ADAR1p110 are variable. Recent findings that ADAR1 is involved in the recognition of self versus non-self dsRNA provide potential explanations for its links to hematopoiesis, type I interferonopathies, and viral infections. Editing in both coding and noncoding sequences results in diseases ranging from cancers to neurological abnormalities. Furthermore, editing of noncoding sequences, like microRNAs, can regulate protein expression, while editing of Alu sequences can affect translational efficiency and editing of proximal sequences. Novel identifications of long noncoding RNA and retrotransposons as editing targets further expand the effects of A-to-I editing. Besides editing, ADAR1 also interacts with other dsRNA-binding proteins in editing-independent manners. Elucidating the disease-specific patterns of editing and/or ADAR1 expression may be useful in making diagnoses and prognoses. In this review, we relate the mechanisms of ADAR1′s actions to its pathological implications, and suggest possible mechanisms for the unexplained associations between ADAR1 and human diseases.

## 1. Introduction

Post-transcriptional modification of RNA is a well-known mechanism of expanding the transcriptome and the range of functions of RNA transcripts. Among the various forms of post-transcriptional modification is the deamination of adenosine to form inosine, in a reaction known as A-to-I editing. This reaction is catalyzed by a family of enzymes named Adenosine Deaminases Acting on RNA (ADAR). ADAR, first identified as an RNA unwindase [1,2], was later found to also edit double-stranded RNA (dsRNA) [3,4]. Since its discovery, much has been learned about its functions in gene regulation and its implications in development and diseases, and these functions can be either editing-dependent or independent. This review focuses on the current knowledge about ADAR1, the most prevalent form of ADAR, and its roles in human diseases.

### 1.1. Mechanism of A-to-I Editing

**Figure 1:**
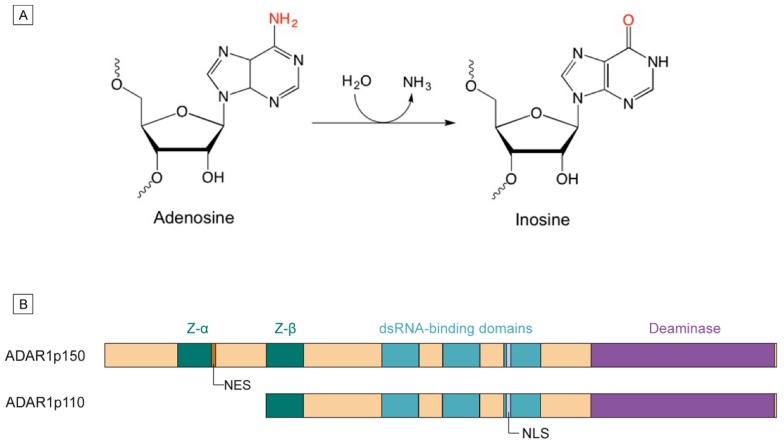
Catalytic function and isoforms of Adenosine Deaminase Acting on RNA 1 (ADAR1). (**A**) ADAR1 catalyzes the hydrolytic deamination of adenosine to form inosine, which is recognized by cellular machineries as guanosine; (**B**) Gene structures of the two isoforms of ADAR1. Z: Z-DNA binding domain; NES: nuclear export signal; NLS: nuclear localizations signal.

ADARs target double-stranded RNA formed both intramolecularly and intermolecularly. Editing occurs via the hydrolytic deamination at C6 of adenosine, changing it to inosine (Figure 1A). Cellular machineries read inosine as guanosine, making the effect of A-to-I editing similar to an A-to-G substitution. This expands the transcriptome when editing occurs in coding mRNA sequences, in start and stop codons, or at splice sites. However, most known ADAR substrates are noncoding sequences, like microRNAs (miRNAs) and the dsRNA formed from inverted Alu repeats. Binding and editing are generally not sequence-specific [5], though ADAR1 [6] and ADAR2 [7] appear to have distinct 5′ and 3′ neighbor preferences in their editing sites, respectively. Instead, the dsRNA structure appears to be more important in ADAR’s recognition of its substrates [8,9].

### 1.2. ADAR1 Isoforms and Domain Structures

The general structure of ADAR enzymes involves two to three dsRNA binding motifs, and a catalytic deaminase domain at the C terminus. In vertebrates, three types of ADARs have been identified, named ADAR1 [10], ADAR2 [11], and ADAR3 [12,13]. ADAR1 was the first to be identified and is the most widely expressed form [10]. Due to the alternate use of promoters and alternative splicing, two isoforms of ADAR1 exist in cells (Figure 1B). Both isoforms of ADAR1 have three dsRNA binding domains (dsRBD), among which the third dsRBD contains the nuclear localization signal (NLS). The interferon-inducible full-length isoform, ADAR1p150, contains a nuclear export signal (NES) in the N-terminal Z-DNA binding domain, causing ADAR1p150 to be mainly localized in the cytoplasm [14], though the presence of a NLS allows it to undergo nucleocytoplasmic shuttling (Figure 1B). The shorter isoform, ADAR1p110, is constitutively expressed. It differs from ADAR1p150 at the N-terminus by a Z-DNA binding domain [15] (Figure 1B), and is mostly localized in the nucleus [16,17]. Due to their expression patterns, ADAR1p150 is often associated with interferon responses, whereas ADAR1p110 may be more important in maintaining normal activity. While this review focuses on ADAR1, ADAR2 has been comprehensively reviewed elsewhere [18]. Unlike ADAR1 [10] and ADAR2 [11], ADAR3 has no known editing activity [19].

## 2. ADAR1′s Role in Immunity

The requirement of ADAR1 for life was first recognized from the embryonic lethality of *Adar1*^−/−^ in mice by embryonic day 12.5 (E12.5), with substantial overproduction of interferon and widespread apoptosis [20]. Death of embryonic liver hematopoietic cells and liver disintegration were also observed, implicating ADAR1 in hematopoiesis and organ development as well. Furthermore, editing by ADAR1 was observed in immune organs and lymphocytes in response to inflammation, which can be triggered by external sources, like viruses, as well as internal sources, as in autoimmune diseases [21]. These suggested early on that ADAR1 may have a role in regulating inflammatory and immune responses.

The cause of such embryonic lethality and inflammation was not well understood until the last few years, with the elucidation of ADAR1′s role in immunity. One interesting idea is that editing of RNA by ADAR1 differentiates “self” RNA from “non-self” RNA, where the non-self RNA can be exogenous or endogenous. Retinoic acid-inducible gene I (RIG-I)-like receptors (RLRs), including melanoma differentiation-associated protein 5 (MDA5) and RIG-I, are cytosolic RNA surveillance machineries that screen for pathogenic material. Both these RLRs interact with the mitochondrial activation signaling (MAVS) protein, ultimately activating transcription factors that initiate the expression of immune response genes, ranging from interferon (IFN) to antiviral genes [22].

**Figure 2:**
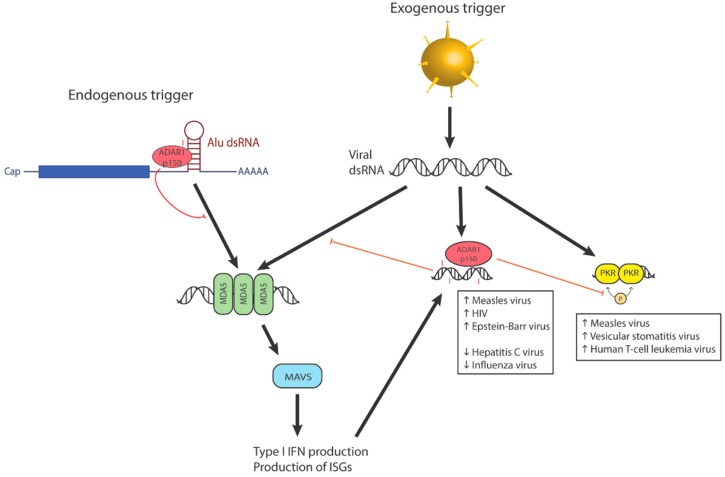
Editing-dependent and editing-independent effects of ADAR1p150 on the interferon pathway. Being a product of an interferon-stimulated gene, ADAR1p150′s expression is also upregulated by the interferon pathway. Besides exogenous sources of dsRNA, the innate immune system can also be triggered by endogenous dsRNA, such as that formed between inverted repeats of Alu sequences at the 3′ UTRs of coding sequences.

The RIG-I family of proteins detect foreign RNA and activate signaling responses that result in IFN production. While one group suggested that binding of ADAR1 to dsRNA may affect the accessibility of RIG-I to the same dsRNA substrate [23], others have suggested that only MDA5 is involved in ADAR1’s differentiation between self and non-self RNA [24]. ADAR1 specifically regulates the MDA5-MAVS pathway, which senses the dsRNA formed during viral replication [24] (Figure 2). Liddicoat et al. suggested a mechanism whereby the I:U mismatches generated by editing could prevent MDA5 oligomerization and its subsequent activation [25]. However, the preference of MDA5 for edited dsRNA substrates could also inhibit its activation by other unedited transcripts [25]. In effect, editing may suppress potential downstream IFN responses, and reduce the undesired autoimmune responses to unedited endogenous dsRNA.

A more recent study found that A-to-I editing, specifically by the p150 isoform, is required to prevent innate immune responses (Figure 2), manifested as the formation of stress granules and eIF2-α phosphorylation following interferon induction. George et al. proposed that the role of A-to-I editing by ADAR1 in innate immunity may simply be to destabilize the dsRNA, and thus downregulate the activation of protein kinase R (PKR), RIG-I and MDA5. On the other hand, the absence of ADAR1 would allow dsRNA to accumulate above the threshold required for activation of these cytoplasmic dsRNA sensors [26].

However, besides the observation that ADAR1 regulates the sensing of dsRNA by MDA5, the reverse also occurs, whereby detection of dsRNA in the cytosol triggers the production of ADAR1p150, paralleling the induction by IFN [27] (Figure 2). Thus, rather than a one-way relationship, ADAR1 may instead be part of a regulatory feedback loop with RLRs, exhibiting an intricately balanced mechanism to control the immunogenic effects of dsRNA in the cytosol.

### 2.1. Hematopoiesis

Knockout of ADAR1 in mice causes the loss of embryonic liver hematopoietic cells [28], and fetal liver-derived hematopoietic stem cells require ADAR1 for survival [29]. Hartner et al. suggested that ADAR1 plays an important role in the survival of hematopoietic stem cells (HSCs) as they progress to the multipotent progenitor stage [29]. In the absence of ADAR1, the hematopoietic defect due to hyper-proliferation may have resulted from the failure to regulate interferon signaling [29]. However, it was later reported that ADAR1 is involved in the regulation of differentiating hematopoietic progenitor cells, and not the more primitive cells such as HSCs [30], contradicting Hartner et al.′s study. Additionally, the synthesis and maintenance of mature blood cells are also disrupted in *Adar1*^−/−^ mice, and these effects are likely to be editing-dependent.

Several mechanisms have been proposed to explain the involvement of ADAR1 in hematopoiesis. Editing may occur at the long 3′ untranslated regions (3′ UTRs) of three genes that are either overexpressed or underexpressed in the erythroid lineage [25]. Embryonic lethality of *Adar1*^−/−^ mice may also be due to the aberrancy of immune responses and IFN signaling, triggered by the formation of undesired dsRNA complexes. In *Adar1*^−/−^ mice, additional mutations that inhibit the activation of antiviral systems, such as IFN response pathways, appeared to delay their death. Yet, the largest effect was seen in *Mavs*^−/−^:*Adar1*^−/−^ double knockouts [31]. In hematopoietic stem (HSC) and progenitor (HPC) cells, MDA5 is the key receptor that detects the presence of unedited dsRNA [22]. ADAR1 may suppress autoimmune responses in HSCs and/or HPCs by editing endogenous dsRNA, such that the excess of unedited dsRNA in ADAR1’s absence results in stem cell apoptosis. However, MAVS may also be involved in apoptosis in manners independent of transcription [24]. While the editing targets were not identified, ADAR1 had to be catalytically active to inhibit the aberrant immune responses in the MDA5-MAVS pathway. In this case, both isoforms of ADAR1 appeared to be important.

The involvement of ADAR1 in the MDA5-MAVS immune pathway may explain the dysregulation of interferon signaling in *Adar1*^−/−^ knockout cells, which was proposed to lead to hematopoietic defects [29]. However, Pestal et al. suggested that the MDA5-MAVS pathway only has an indirect effect on hematopoiesis, and the true pathway may be MDA5-MAVS-independent, especially in the case of post-natal mortality [24]. ADAR1p110 was found to be insufficient to sustain B cell development, and ADAR1p150 may be more strongly implicated here [24].

### 2.2. Type I Interferonopathies

The implication of ADAR1 in immunity would also explain several autoimmune diseases, termed Type I interferonopathies [32], that have been associated with mutations on ADAR1 itself. Aicardi-Goutières syndrome (AGS) is a fatal childhood encephalopathy characterized by uncontrolled expression of the antiviral cytokine IFN-α, giving rise to symptoms reminiscent of a viral infection [33]. Another well-studied disease that arises from mutations on ADAR1 is dyschromatosis symmetrica hereditaria (DSH), a mild genodermatosis that results in phenotypes like hyper-pigmented and hypo-pigmented macules on the dorsal parts of one′s hands and feet, as well as freckle-like macules on the faces of infants and young children from Japan and China [34]. Kondo et al. have suggested that DSH may be an interferon-induced condition [35]. Bilateral striatal necrosis (BSN), a dystonic or rigid movement disorder arising from abnormalities in the brain, also occurs with an upregulation of interferon stimulated genes [36]. One case of spastic paraplegia, another hereditary disease involving axonal degeneration and lower limb spasticity, was also associated with mutations on ADAR1. In both BSN and spastic paraplegia, however, patients do not show characteristic symptoms of AGS [37].

AGS, DSH, BSN and spastic paraplegia patients who bear ADAR1 mutations all share one common G1007R substitution, which completely abolishes editing [31]. A possible mechanism is that the G1007R mutant could bind more tightly to RNA, thereby preventing correct RNA editing [38]. A few other amino acids, like R892 and Y1112 in the deaminase domain, are also mutated in two or more of the four diseases, either by missense or nonsense mutations. Another recurrent mutation, the P193A mutation, occurs in the Z-α DNA/RNA binding domain at the N-terminus of the p150 isoform [38]. This, coupled with the finding that the G1007R mutation has a more detrimental effect when located on ADAR1p150 [31], implicates ADAR1p150 specifically in AGS and BSN. In general, only nine mutations on the ADAR1 coding sequence have been identified in AGS patients, compared to the more than 130 amino acids that have been found to be mutated in DSH patients. In addition to Hayashi et al.′s list of known mutations [39], novel mutations in ADAR1 that are associated with DSH have also been identified [40,41,42,43,44,45], including an in-frame insertion that leads to mis-splicing [43].

The observation that the disease genotypes are sometimes monoallelic supports haploinsufficiency as a mechanism in DSH. This resembles the finding that in *Adar1*^+/−^ heterozygotic mouse embryonic fibroblasts, the level of ADAR1, rather than its editing activity, was the stronger determinant of the same aberrant immune response that gave rise to embryonic death in *Adar1*^−/−^ mice [31]. Alternatively, given that ADAR1 homodimerization seemed necessary for its catalytic function [46], and given the catalytically nonfunctional heterodimer formed between the mutant ADAR1 lacking RNA binding abilities and wildtype ADAR1 [47], another explanation could also be the dominant negative effect exerted by the mutant ADAR1, by binding to wildtype ADAR1 [38]. In contrast, both homozygous and heterozygous cases of ADAR1 mutants have been identified in AGS patients, and one standing hypothesis is the hypomorphic nature of these mutants. There also seems to be a distinction of ethnicity, with DSH patients being East Asian [39], and AGS patients being mostly Caucasian, but also Pakistani, Indian and Brazilian [38]. Similarly, patients with BSN associated with ADAR1 mutations were mostly White British [36], while the single patient with spastic paraplegia was European American [37].

With the finding that ADAR1 suppresses interferon responses indirectly by regulating the sensing of dsRNA by MDA5, it may be that wildtype ADAR1 serves to edit endogenous RNA transcripts, whereas this editing is reduced in patients with type I interferonopathies. The accumulation of unedited dsRNA may more actively trigger the MDA5-MAVS pathway, leading to stronger, more unregulated interferon responses. Interestingly, another mutation associated with spastic paraplegia is the IFIH1 gene that encodes MDA5 [37], strongly supporting the role of dsRNAs in such diseases. In general, the regulation of nucleic acids appears to be the key in unifying these diseases, and where ADAR1 is concerned, its regulation of dsRNAs may represent a potential path to diagnoses or therapies. The particular dsRNA involved, whether it be retroelements [32] or other forms, still remains an open question.

## 3. Editing of Exogenous Viral dsRNA

Besides endogenous sources of dsRNA, exogenous viral dsRNA can also trigger immune responses via the RIG-I or MDA5-MAVS pathway. The theory that ADAR1 could serve a protective function against viral infections was prompted by the early observation that ADAR1 both edits and unwinds dsRNA [4]. Double-stranded RNA is produced by many types of viruses at some point in their lifecycle, creating the potential for ADAR1 to regulate the interactions between viral RNA and host cellular machinery. Furthermore, ADAR1 was found to be vital in the suppression of type I IFN responses in cells, and the finding that synthetic dsRNA containing inosine, such as consecutive I:U wobble base pairs, suppresses interferon responses [48] drove the belief that there could be a functional relationship between the level of editing in exogenous dsRNA and the host cell responses to these dsRNA.

### 3.1. Editing Affects Viral Recognition by RLRs

RLRs recognize foreign dsRNA as a defense mechanism against viruses. A recent study identified defective interfering RNA (DI-RNA) in measles viruses, which arises from the premature termination of replication and which can form dsRNA hybrids with the template genome. Biased hypermutations of A-G and U-C in DI-RNA sequences corresponded with editing by ADAR1. Unedited double-stranded DI-RNA can trigger innate immune responses by activating proteins like MDA5 and RIG-I, while edited DI-RNA may be destabilized, allowing measles virus to escape immune detection [49] (Figure 2).

However, mouse embryonic fibroblasts with a deletion for ADAR1p150 became more susceptible to measles virus infection, suggesting an editing-dependent, antiviral role of ADAR1 too. This mechanism may be common among members of the *Paramyxoviridae* family, and even all respiratory RNA viruses [50]. Like measles virus, ADAR1p150 was also found to protect hosts against infection by the influenza A virus. The single stranded RNA (ssRNA) of influenza A virus is in fact a target of A-to-I editing, probably via a dsRNA intermediate, and this edited ssRNA promotes Toll-like receptor 7 (TLR7) sensing and subsequent synthesis of IFN-α [51]. Hence, ADAR1 also counters infection by influenza A virus by promoting the activation of interferon response (Figure 2). It is plausible that the measles virus, which also has an ssRNA genome, may share a similar mechanism.

### 3.2. Editing on Viral Coding Sequences Affects Viral Life Cycles

The viral dsRNA of hepatitis D virus (HDV) was the first to be identified as an editing viral substrate of ADAR1, with a specific editing site. ADAR1 targets HDV’s anti-genome RNA within the stop codon of the short delta antigen (HDAg-S)′s open reading frame. Creation of the amber/W site disrupts termination and results in a longer protein product, the long delta antigen (HDAg-L) [52]. HDAg-S functions in viral RNA replication, whereas HDAg-L is required for virion morphogenesis, but prevents replication. In effect, this switches the viral life cycle from proliferation to packaging into non-infectious virions. Although both ADAR1 and ADAR2 were found to edit the amber/W site on the HDV anti-genome, it was posited that ADAR1 may be the main editor at play, due to its higher expression levels. In particular, the p110 isoform is likely responsible for this editing [53].

In subacute sclerosing panencephalitis (SSPE), a fatal disease where the measles virus infection is localized in the brain, the viral genome contains biased mutations of A-G and U-C, due to editing by ADAR1. These editing sites were found mostly in the RNA of the M gene, which codes for the matrix protein. These mutations inhibit the production of the matrix protein, resulting in reduced viral assembly and release, and ultimately persistent infection [50].

Infection by hepatitis C virus gives rise to chronic liver disease, cirrhosis, and hepatocellular carcinoma. In Huh7 hepatoma cell lines, ADAR1 levels and A-to-I editing were upregulated upon IFN-α treatment. Only ADAR1p150 is implicated in this disease development. By diminishing the amount of replicon RNA, A-to-I editing directly inhibits viral replication (Figure 2), possibly via degradation of the edited RNA by inosine-specific RNases [54].

In contrast, editing in the Epstein-Barr virus (EBV) genome facilitates viral release. EBV, a member of the herpes family, causes diseases like infectious mononucleosis and cancers like Hodgkin’s lymphoma. A-to-I hyperediting was found in the OriP transcripts, which are expressed upon induction and entrance into the lytic stage. The leftward transcripts, OriPtL, also interact with ADAR1 during lytic conversion, and the direct recruitment of ADAR1 may be an essential component in the EBV lytic pathway [55] (Figure 2). In addition, EBV is also regulated at the level of miRNAs, which is described in Section 5.1.

However, there have been several conflicting reports on the effects of editing in human immunodeficiency virus 1 (HIV-1). Editing of the HIV-1 RNA, as observed in primary macrophages, may inhibit viral propagation by disrupting viral assembly. In HIV-1/tuberculosis co-infected patients, viral infection induced ADAR1 to edit the RNA transcript of the HIV-1 envelope glycoprotein, possibly targeting the HIV-1 RNA for degradation [56]. In human primary CD4+ T cells, however, the increased expressions of both ADAR1 isoforms were concurrent with an enhanced HIV-1 viral replication [57], due to augmented viral protein synthesis [58] (Figure 2). This was attributed first to the increased expression of the p24 Gag protein [59]. A-to-I editing was later identified in the noncoding 5′ UTRs and coding regions of Rev and Tat mRNA, notably leading to nonsynonymous amino acid changes in Rev. The effects of editing, however, were not elucidated [60]. More recently, ADAR1 was also found to be packaged into HIV-1 virions, via interactions with the p55 Gag protein, and these interactions may be bridged by cellular RNA [61].

### 3.3. Editing-Independent Interactions of ADAR1 with Other Interferon-Stimulated Proteins

Besides its catalytic function, ADAR1 also serves other roles that are apparently independent of editing, by interacting with other interferon-inducible proteins that bind dsRNA. For instance, activated PKR leads to downstream antiviral effects, including stress granule formation and apoptosis. ADAR1p150, produced in response to viral infections, inhibits PKR activation by blocking phosphorylation [62] (Figure 2). This inhibition was observed in measles virus [63], vesicular stomatitis virus [22], human T-cell leukemia viruses 1 and 2 (HTLV-1 and HTLV-2) [57], and HIV-1 [60]. The antagonization of PKR activation by ADAR1 requires its dsRNA binding function, but not necessarily its editing function [57]. In these cases, the interferon-inducible ADAR1p150 seems to disrupt the interferon response [57,63,64].

In contrast, ADAR1p150 may interact with nuclear factor 90 (NF90) to promote antiviral responses [65]. This interaction, bridged by dsRNA [65], has been proposed to enhance the expression of genes that are under the control of NF90, such as the IFN-β gene [66].

## 4. Editing of Coding Endogenous dsRNA

For editing to occur in coding sequences, premature mRNA has to fold back to form an imperfect dsRNA structure with the intronic, editing-site complementary sequence [19]. RNA editing appears to be particular to both cell type and cell environment, and thus ADAR1′s impact cannot be easily generalized. Both hyper- and hypo-editing of coding regions can contribute to diseases, which are most commonly cancers. Besides cancers, editing of mRNA in neurons also brings into the picture some neurological conditions that are controlled by ADAR1.

### 4.1. AZIN1

Antizymes inhibit cell growth by facilitating the degradation of cell cycle regulatory proteins, like cyclin D1. On the AZIN1 mRNA, which codes for antizyme inhibitor 1, editing results in a nonsynonymous substitution from serine to glycine [63]. Editing of the AZIN1 mRNA stabilizes the AZIN1 protein, increasing its binding to antizymes and preventing the degradation of oncoproteins. Two cancers associated with increased editing on AZIN1 are hepatocellular carcinoma (HCC) and esophageal squamous cell cancer (ESCC) [67]. Based on relative expression levels, editing is believed to depend only on ADAR1. In both cancers, hyper-editing may partly be attributed to the higher levels of ADAR1. ADAR1p110 appears to be responsible, although no direct comparison was made with ADAR1p150. Notably, while ADAR1 promotes cancer cell proliferation, ADAR2 instead has a tumor-suppressive function [68]. Furthermore, editing in AZIN1 also decreases the sensitivity of the edited protein to targeted agents, implicating RNA editing in therapeutic efficacy as well [69].

### 4.2. FLNB

In primary ESCC, another target of editing is the mRNA of filamin B (FLNB) [67]. Filamin B is usually used for the construction of the cytoskeleton, but hyper-edited FLNB mRNA is oncogenic. In vitro, uncontrolled hyper-editing by both ADAR1p110 and ADAR2 contributes to abnormal cell proliferation [68]. In primary ESCC tumors, hyper-editing may similarly be due to higher ADAR1 levels, and this overexpression could be attributed to gene amplification of ADAR1 [67] on chromosome 1q21 [70]. 

### 4.3. NEIL1

Similarly, the increased level of editing observed in human non-squamous cell lung carcinoma (NSCLC) cell lines may be attributable to increased editing activity, or simply to the larger amount of ADAR1 from gene amplification. Of note is the editing of NEIL1 mRNA, whose protein product is involved in DNA base excision repair [71]. Editing of NEIL1 was previously found to modify the specificity and efficiency of DNA repair, which corroborates with the IFN-inducible nature of ADAR1p150 [72]. It would be interesting to look into the effects of the edited NEIL1 on cancer-promoting or cell cycle regulatory proteins in NSCLC cell lines.

### 4.4. GLI1

Glioma-associated oncogene 1 (GLI1) is a transcription factor that functions at the end of the Hedgehog signaling pathway, and its upregulation is associated with basal cell carcinoma in mice and cerebellar tumor formation in humans. A-to-I editing in the coding region of GLI1 results in an R701G substitution, which is predicted to affect the protein structure. The fact that editing is more prevalent in normal cells than in tumor cells suggests that editing may reduce the oncogenic potential of GLI1. Besides ADAR1, ADAR2 has also been found to edit GLI1 [73].

### 4.5. PTPN6

Editing of the hematopoietic cell tyrosine phosphatase PTPN6 transcript is linked to acute myeloid leukemia (AML) [74], but editing does not occur in the subgroup of AML with normal karyotype [75]. In the former case, higher levels of editing may inhibit post-transcriptional processing of the phosphatase, ultimately allowing growth receptors to proliferate without regulation [76]. However, it is not clear whether ADAR1 or ADAR2 is responsible for this editing activity.

### 4.6. GABRA3

The chloride channel, GABRA3, is both necessary and sufficient for breast cancer invasion and metastasis [77]. A-to-I editing on the GABRA3 transcript results in a single amino acid change in the α3 protein, at the I/M site [78], which reduces the metastatic potential of breast cancer cells. Mechanistically, the edited GABRA3 protein inhibits Akt activation, and thus cell motility, invasion and metastasis. Additionally, intracellular transport of the edited GABRA3 protein is modified, disrupting its localization on cell membranes. Only ADAR1p110 was identified in the non-invasive, editing-active cell lines, hence implicating this isoform in the prevention of breast cancer [77]. Interestingly, an intronic duplex downstream of the exonic hairpin structure, where the I/M editing site is located, appears to be required for editing to occur, raising the possibility that editing may also be cis-regulated by distant elements [79].

### 4.7. RHOQ

A-to-I editing of the Ras homolog family member Q (RHOQ) transcripts gives rise to an N136S substitution [80]. N136 is required for interactions between RHOQ and other proteins, although the interactions that are affected by the N136S substitution are unclear at this point. This substitution causes the actin cytoskeleton to rearrange, and the cell to become more invasive, but has no effect on the rate of proliferation. Gastric cancer, NSCLC and colorectal cancer have been correlated with an increased amount of editing of RHOQ transcripts. Studies using clinical samples of colorectal cancer cells provided additional support for the relationship between editing on *RHOQ* and the aggressiveness of the cancer [80].

### 4.8. CAPS1

A new target for ADAR1 was identified recently in the human *CADPS* gene, resulting in a E1250G substitution [81]. The encoded CAPS1 protein belongs to a family of proteins that regulate the exocytosis of dense-core vesicles, which are formed during the secretion of insulin and neurotransmitters. The E1250G mutation causes increased catecholamine secretion. While this mutation has been shown in mice to result in increased locomotion and leaner bodies, the CAPS protein family has also been implicated in autism and learning disabilities, and further studies are needed to determine if editing by ADAR1 also affects the development of these disorders [81].

### 4.9. HTR2C

The HTR2C transcript, which encodes serotonin receptor 2C, contains several editing sites, among which two are edited mainly by ADAR1 [24,28]. In mice, over-editing results in metabolic imbalances, such as decreased fat accumulation, increased energy expenditure, and a compensatory increase in appetite [82]. In clinical samples, suicide victims with major depressive histories also exhibit the same editing sites, together with an increase in ADAR1 levels in their cortices [83]. Furthermore, ADAR1p110 appeared to be both necessary and sufficient for editing at both these sites [24]. A more recent investigation suggested that in victims of suicide, receptor hypoactivity due to editing results in a dysregulation of the expressions of other genes related to synaptic transmission [84], as well as increased susceptibility to cocaine dependence [85].

### 4.10. GRIA2

The R/G site on the AMPA receptor subunit encoded by GRIA2 is edited by both ADAR1 and ADAR2. Edited GRIA2 recovers more quickly from desensitization, resulting in enhanced synaptic responses [86,87]. Editing was increased by 20% in the hippocampal tissue of epilepsy patients [88], whereas acute spinal cord injury appeared to decrease the extent of editing, possibly to compensate for the glutamate excitotoxicity from the injury [89]. In the latter case, increased editing was believed to be an adaptation specific to GRIA2, rather than a general increase in editing activity on all substrates [89]. Treatment with the GABA-A channel blocker, bicuculline, appeared to promote R/G editing in CA1, but not CA3 hippocampal cells [90], but this cell-specific response was contradicted by a later finding that chronic glutamate treatment reduced the R/G editing levels in GRIA2 in primary cortical neurons [91]. In this later study, lower R/G editing levels were correlated with lower ADAR1 and ADAR2 levels [91]. The observation that calpain, induced by the glutamate treatment, can cleave ADAR2 [92] raises the possibility that a similar mechanism may also occur to ADAR1 [91]. In contrast to AZIN1, editing on GRIA2 may increase its sensitivity to therapeutic agents [69].

Besides GRIA2, the coding sequences for other glutamate receptors like GRIA3 [86], GRIA4 [86], GRIK1 [93] and GRIK2 [94,95] are also editing targets of both ADAR1 and ADAR2. Unlike editing in GRIA3 and GRIA4 that affects their recovery from desensitization, as in GRIA2, editing in GRIK1 and GRIK2 instead affects calcium channel permeability.

### 4.11. Comparing ADAR1′s and ADAR2′s Substrates

While ADAR1 and ADAR2 generally have distinct substrates due to their different mechanisms for substrate selection [96] and different structures of the dsRNA-binding domains [97], there are some instances where they share the same substrate, like FLNB, GRIA2, and HTR2C. ADAR1 and ADAR2 share similar editing site preferences, such as an A:C mismatch [98], conceivably due to the gain in stability that results from editing, though it is also possible that the active sites can better accommodate cytosine [98]. The two enzymes also show similar preferences for the nucleotide 5′ to the editing site [9], but only ADAR2 has a 3′ neighbor preference [9]. On the structural level, both enzymes also appear to prefer imperfectly base-paired dsRNA that contains internal loops, which further restricts the potential substrates and the modes of binding [9]. It is noteworthy that the shared editing sites are usually in coding regions, which tend to be shorter than their noncoding counterparts. Given that a minimum dsRNA length is required for ADARs to bind and catalyze editing [99,100], length may be one factor that restricts both enzymes to the same target sites. Crystal structures of ADAR2 revealed that specific helical structures are recognized by ADAR2’s dsRBDs [8], and while such studies have not been done on ADAR1, similar explanations may also be applicable to ADAR1.

## 5. ADAR1′s Effects on MicroRNA

MicroRNA (miRNA) is a type of noncoding RNA that is first transcribed as a hairpin structure, named primary miRNA (pri-miRNA). In the maturation process, pri-miRNA is cleaved by the Drosha/DGCR8 complex into precursor miRNA (pre-miRNA), which is then further cleaved by Dicer in the cytoplasm. Mature miRNAs are single-stranded structures that downregulate the level of messenger RNAs, facilitated by the RNA-induced silencing complex (RISC) [101]. ADAR1 can affect the process of miRNA-mediated regulation of gene expression in both editing-dependent and -independent manners. The widespread functions of miRNA mean that ADAR1 may also be implicated indirectly in many cellular pathways, from development to oncogenesis. In most cases, the p110 isoform is associated with pri-miRNA levels and the p150 isoform with pre-miRNA levels, likely due to their different subcellular localizations [102].

### 5.1. Editing of miRNA

**Figure 3:**
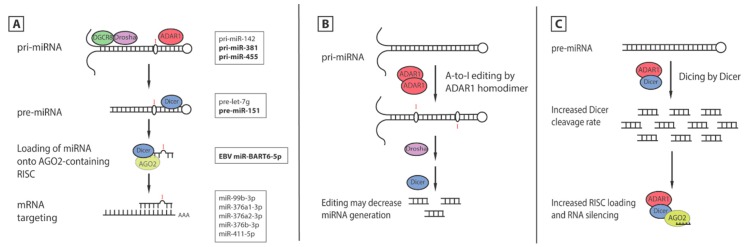
Effects of ADAR1 on miRNA synthesis and activity. (**A**) Steps in miRNA synthesis where editing by ADAR1 has effects are indicated. Bolded miRNAs are associated with diseases discussed in this review; (**B**) Editing by ADAR1 homodimer on pri-miRNA may decrease the rate of miRNA processing by Drosha and Dicer, resulting in reduced amounts of miRNA and decreased mRNA silencing; (**C**) Alternatively, ADAR1 may form a heterodimer with Dicer, and upregulate its dicing activity. This interaction also increases loading of miRNA onto RISC, resulting in increased mRNA silencing.

One of the earliest studies on editing of miRNAs suggested that editing may affect their processing [82]. Editing of miRNAs has been increasingly associated with cancers, and its effects can be either oncogenic or tumor-suppressive [103]. For example, the unedited pri-miR-455-5p promotes melanoma by silencing the expression of the tumor suppressor CPEB1 in metastatic melanoma, gastric, thyroid and lung cancers, while editing is tumor-suppressive (Figure 3A). Bioinformatics studies revealed that other potential target sites of miR-455-5p include the transcripts of RHOC, MDM4 and integrin α-2 [104], all of which facilitate tumor growth. Some cancers linked to these regulatory genes include pancreatic carcinoma [105], head and neck squamous cells [106], breast cancer [107], cutaneous melanoma [108], and retinoblastoma [109]. Editing may decrease the efficiency of processing by the Drosha/Dicer system (Figure 3B). Structural modifications resulting from editing may also affect the accessibility of Drosha to pri-miRNA [104]. However, ADAR1 may also be physically involved in the complex formation between miR-455 and Drosha or Dicer.

In contrast, in vivo studies showed that increasing the expression levels of edited miR-381 is correlated with increased lung cell proliferation [71] (Figure 3A). miR-381 was found in several studies to be involved in pathways towards cancer, such as stemness [110] and chemoresistance [111]. Hence, in the case of lung cancer, A-to-I editing by ADAR1 increases the likelihood of cancer development.

Other than cancers, editing on miRNA may also be implicated in development. For instance, miRNA-151 was found at significantly lower levels in mouse oocytes and zygotes, but the higher levels of primary miR-151 suggested that the point of control could lie in miRNA processing and maturation (Figure 3A). One hypothesis is that the increased rate of A-to-I editing in maternally inherited pri-miR-151 could direct it towards degradation [112]. An alternative explanation could be derived from an earlier finding that editing of pre-miR-151 inhibits its cleavage by the Dicer-TRBP complex, and thus its downstream processing [113]. High levels of the p110 isoform suggested that ADAR1p110 alone may be sufficient for this regulation. This reprogramming by ADAR1’s catalytic activity could thus be an essential aspect of developmental control [112].

Besides endogenous targets, ADAR1′s effects on miRNA have also been observed in EBV. Of the four miRNA targets identified in EBV, miR-BHRF1-I, miR-BART6, miR-BART8 and miR-BART16, editing of pri-miR-BART6 inhibits the miRNA processing pathway by suppressing RISC loading (Figure 3A). Ordinarily, miR-BART6-5p has inhibitory effects on Dicer, possibly leading to a global downregulation in the host cell′s miRNA synthesis, and disrupting miRNA-mediated pathways that may regulate immune responses against viral infections. Hence, contrary to the editing of the OriP transcripts, editing of EBV′s miRNA by ADAR1 may have antiviral effects, by preventing the maturation of miR-BART6 [114].

### 5.2. Editing of Seed Sequence Changes mRNA Targets

On the relatively rare occasion that A-to-I editing occurs in the seed sequence of miRNA, or that editing occurs in the 3′ UTRs that serve as miRNA binding sites, the targeting of mRNAs by miRNAs would be affected. Modified seed sequences were observed in pri-miR-376a [115], for instance, resulting in a change in the target mRNA. While only ADAR2 is responsible for editing pri-miR-376a, one could speculate similar mechanisms in ADAR1′s pri-miRNA targets as well.

### 5.3. Editing-Independent Effects of ADAR1 on miRNA Processing

Given the interactions of ADAR1 with enzymes involved in miRNA processing, ADAR1 may affect miRNA synthesis in editing-independent ways. That ADARs may inhibit miRNA processing was first observed in ADAR2 [116]. Later, binding of miRNA was also found to directly affect its accessibility to the miRNA processing proteins like Drosha and Dicer [117]. A clear case of ADAR1′s steric effects involves its binding to pri-miR-302, whose mature miRNA is depleted during human embryonic stem cell (hESC) differentiation. Binding of ADAR1 prevents DGCR8 dimers from accessing the RNA, thus inhibiting Drosha cleavage, suggesting that ADAR1 may be indirectly involved in hESC differentiation by regulating miRNA biogenesis [118]. However, another group proposed that the effect of ADAR1 on Dicer is not direct, but instead through a bridging dsRNA, such as let-7 or other primary miRNAs [102]. Another suggestion is an interaction between ADAR1 and DGCR8, instead of Dicer or Drosha, such that ADAR1 physically inhibits the required interaction between DGCR8 and Drosha [102]. The downregulation of miRNA processing by ADAR1 may be a required process for inhibiting neural induction and differentiation of hESCs, by regulating the levels of let-7 [118]. In cancers, ADAR1 appears to decrease the levels of miR-221 and miR-222, which target the cell surface glycoprotein ICAM1 gene and whose overexpressions are involved in cancers like pancreatic cancer, papillary thyroid carcinoma, prostate carcinoma, and metastatic melanoma [119]. The positive correlation between increased ADAR1 levels and reduced apoptosis is reminiscent of the embryonic lethality of *Adar1*^−/−^ mice, although when applied to cancer cells, high ADAR1 levels would be harmful to the host organism.

However, Ota et al. proposed that ADAR1 might increase miRNA production [120] (Figure 3C). While ADAR1 homodimers can edit dsRNA [46], ADAR1 may also form heterodimers with Dicer to affect the processing of miRNA, independent of its editing activities [120]. Both isoforms interact with RISC proteins and Dicer, even without an active deaminase domain. In addition, the ADAR1-Dicer heterodimer also interacts with AGO2. By binding to Dicer, ADAR1 was found to promote Dicer’s activity by speeding up miRNA and siRNA processing, and to upregulate RISC assembly and loading. Significantly, ADAR1 appeared to function like Dicer′s canonical binding partner, TRBP, and the affinity of ADAR1 for Dicer was just as strong as that of TRBP. Hence, ADAR1 may aid in cellular RNA silencing functions. Ota et al. also proposed an alternative mechanism to explain the embryonic lethality of ADAR1 knockout mice, where increased miRNA population, facilitated by ADAR1, may be necessary for proper embryo development [120].

## 6. ADAR′s Effects on Other Noncoding RNA

### 6.1. Editing of Alu dsRNA

Alu is the most commonly occurring retrotransposon in the human genome, and occurs in tandem repeats. When the repeats are inverted, the resulting RNA transcript can undergo intramolecular complementary base pairing to form double stranded structures, which then serve as substrates of ADAR1. Using a newly developed computational method, Ramaswami et al. confirmed previous findings that a large majority of editing sites are located in Alu sequences, emphasizing the importance of Alu RNA editing [121]. Furthermore, almost all adenosines in Alu repeats are edited, although mostly at low levels [122]. Although the purpose of A-to-I editing in Alu dsRNA is not well understood, several effects of Alu editing may be particularly relevant to diseases. Editing of intronic Alu may result in Alu exonization, which can control mRNA levels and translational efficiency [123,124], and potentially contribute to disease development. On the other hand, base pairing of highly edited intronic Alu repeats can also result in excision of the embedded exon, producing circular RNA (circRNA) [125]. Since circRNA has been thought of as a “miRNA sponge” that traps certain miRNAs [126], the decrease in circRNA production that occurs with Alu editing by ADAR1 [125] may lead to increased amounts of miRNAs, like miR-7, that are associated with cancers [127]. Furthermore, since Alu repeats are often found within the 3′ UTRs of coding regions (Figure 2) where miRNAs bind [128], editing of Alu sequences can potentially change or create new miRNA targets [129].

Alternatively, editing at the 3′ UTR of mRNA may alter its secondary structure and affect its stability. Recently, Stellos et al. proposed a link between editing at 3′ UTR Alu sequences and atherosclerosis, a chronic inflammatory disease where hardened arteries and thickened walls block blood flow. Editing of Alu inverted repeats in the mRNA of the cathepsin S protein, CTSS, may disrupt the double-stranded structure, allowing the human antigen R (HuR) to bind and stabilize the mRNA more easily. Editing hence increases the level of cathepsin S, promoting angiogenesis and atherosclerosis development. The inflammatory nature of the disease suggests that increased levels of ADAR1p150 may aggravate the disease [130]. This may represent a general mechanism that explains the purpose of the ubiquitous editing of Alu.

Furthermore, Daniel et al. proposed that Alu elements may act as decoys for ADAR to edit other RNA segments in their vicinity, as observed with fusion constructs between Alu sequences and human GABRA3 or mouse *Neil1* transcripts. This was also offered as the explanation for the high editing activity at human GLI1′s R/G site, which contrasts with the low editing levels in other primates that do not have Alu repeats [131].

Alu is also implicated in the development of human embryonic stem cells (hESCs) [132]. Alu sequences in undifferentiated hESCs have high levels of A-to-I RNA editing. In contrast, hESCs that are undergoing neuronal and spontaneous differentiation experience lower levels of editing globally. Here, the presence of inosines may be an important factor in cellular regulation, including mechanisms like splicing [132].

Additionally, dsRNAs containing I:U mismatches, which are known to suppress the interferon signaling pathway, resemble Alu dsRNA [48]. Hyper-editing may thus be protective against aberrant immune responses, whereas under-editing of Alu dsRNA may be an endogenous trigger (Figure 2).

### 6.2. Editing Affects Interactions between Intronic lncRNA and Coding mRNA

Long noncoding RNAs (lncRNAs) are large RNA transcripts that are often processed similarly to coding RNA. In cells, they serve regulatory functions by forming protein-RNA complexes with chromatin regulators, as well as by interacting with genomic DNA or RNA [133]. In the latter case, the RNA duplex formed becomes a viable substrate for ADAR1. An example is the prostate cancer antigen 3 (PCA3) RNA, an antisense long noncoding RNA that binds PRUNE2 pre-mRNA. ADAR can bind to this PRUNE2/PCA3 duplex and edit at both the intronic and exonic regions on both strands. Both ADAR1 and ADAR2 appeared to be relevant in this case [134]. Malignant tumor samples have high levels of PCA3 and low levels of PRUNE2, while the reverse applies for benign prostate samples. It would be worthwhile to look into whether A-to-I editing patterns on the PRUNE2/PCA3 dsRNA can be diagnostic of malignancy in human prostate cancer, and whether other lncRNAs can also serve as editing targets for ADAR1.

### 6.3. Editing of LINE1 Retrotransposon

ADAR1 appears to inhibit the retrotransposition of long interspersed element 1 (LINE1), which is the most common autonomous retrotransposon in the human gene [135]. This inhibition may arise from the interaction of ADAR1 with the LINE1 RNA-protein complex. A separate study identified such an interaction in stress granules, though in this case, only ADAR1p150 would be implicated [117]. Although editing seemed dispensable for this inhibition, and was not observed in the LINE1 regions sequenced, it is still possible that LINE1 regions may be edited. Since LINE1-mediated retrotransposition serves as a general mutagen that is implicated in colorectal cancer [136] and NSCLCs [137], among others [138], editing by ADAR1 may be protective against such aberrations.

## 7. Regulation of ADAR1 Expression

The idea that highly controlled ADAR1 expression levels may be important in maintaining normal cellular functions originated from studies on ADAR1 knockout mice. The finding that ADAR1p110 is catalytically active in the form of a homodimer may explain the importance of controlled levels of ADAR1, and the problem of dominant negative effects also comes into play when mutant ADAR1 coexists with wildtype ADAR1. Variations in ADAR1 levels may lead to diseases due to unbalanced levels of editing, but protein-protein interactions that are directly affected by ADAR1 levels should not be precluded.

In general, higher ADAR1 levels have been correlated with several cancers, such as lobular breast cancer [74] and B cell acute lymphoblastic leukemia [102]. Genome-wide studies have also drawn links between higher editing levels and cancers [69,103,139]. In cases like pediatric astrocytoma, the severity of tumors is also linked to an overexpression of ADAR1 [140]. The causal direction is ambiguous, and it is possible that ADAR1p150 is overexpressed in response to interferon production and cellular stress. However, in childhood acute leukemia, only ADAR1p110 was found to be upregulated, while ADAR1p150 was not, providing an instance where interferon response is unlikely the explanation [141].

A possible pathway linking ADAR1p150′s overexpression to cancer was recently proposed for the case of gastric cancer. In gastric cancer, high ADAR1 levels are correlated with increased cell proliferation and metastasis. While one group reported earlier that the higher expression of ADAR1, together with lower ADAR2 levels, led to a “misedited” phenotype [142], another group suggested that ADAR1p150′s function was to promote the mTOR signaling pathway by phosphorylation [143], although whether editing also occurred was unclear. Further studies would be needed to determine how ADAR1p150 regulates phosphorylation of proteins like mTOR, and whether this pathway is generalizable to other cancers that are also associated with imbalances in ADAR1 levels.

ADAR1p150′s levels are typically controlled by interferon induction. In chronic myeloid leukemia, the increased expressions of IFN-γ-R1 and IL-3Rα upregulate ADAR1′s transcription through JAK2, which controls the biogenesis of let-7 miRNA and the self-renewal of leukemia stem cells. A-to-I editing by ADAR1 is needed for the transformation into the malignant pathway, by inhibiting let-7 miRNA biogenesis and promoting the self-renewal of progenitor cells [144]. This would also explain the higher ADAR1p150 levels observed with dysregulated levels of the myeloid transcription factor PU.1 and mis-splicing of the GSK3-β protein, which drives the blastic transformation between the second and third phases of disease progression [145].

While ADAR1p150 is clearly regulated by interferon-related pathways, the regulation of ADAR1p110 levels is only beginning to be revealed. One possible pathway is through the internal ribosome entry site (IRES)-like dependent translation control by PTBP1, which induces ADAR1p110’s expression from the internal start codon on the mRNA sequence of ADAR1p150. While this was observed in gliomas, more studies would have to be done to verify if this is applicable to other cell types [146].

## 8. Conclusions and Future Outlook

With the growing understanding of the functions of noncoding sequences and their impacts on human diseases, the role of ADAR1 in disease development is increasingly appreciated (Table 1). Swift improvements in sequencing technologies have also facilitated bioinformatics studies that reveal the editing targets of ADAR1 (Table 1). Although we are still far from fully understanding the mechanistic aspects of ADAR1′s action, including how the dsRNA-binding domains on ADAR1 are used, the full structure of ADAR1, and the editing targets of ADAR1 that are precluded from sequencing studies, the present knowledge of ADAR1′s impacts in human pathologies may provide new possibilities for treatments and preventive measures. For instance, the levels of RNA editing by ADAR1 could serve as new tools for diagnosis in cancer stem cell-related illnesses. In situations where ADAR1 overexpression contributes to disease progression, as seen in several cancers, or where ADAR1 interacts with other proteins in editing-independent manners, inhibition of ADAR1 could potentially be another strategy in treatment.

## Figures and Tables

**Table 1:** Summary of pathologies associated with ADAR1.

Disease	Disease Classification	ADAR1 Isoform	Editing-Dependent	Target RNA	Regulation	Ref.
Acute myeloid leukemia	Growth receptor proliferation	? *	Yes	PTPN6 mRNA	Inhibition of post-transcriptional processing of phosphatase	[74]
Aicardi-Goutieres syndrome (AGS)	Autoimmune	P150	Yes	Endogenous dsRNA	Interferon; Inhibition of melanoma differentiation-associated protein 5 – mitochondrial activation signaling complex (MDA5-MAVS)	[33]
Atherosclerosis	Inflammation	P150	Yes	Alu dsRNA in CTSS mRNA	Disruption of dsRNA structure	[130]
Bilateral striatal necrosis (BSN)	Autoimmune	P150	Yes	Endogenous dsRNA	Interferon; Inhibition of MDA5-MAVS	[36]
Breast cancer	Metastasis	P110	Yes	GABRA3 mRNA	Inhibition of Akt activation, disruption of localization	[77]
Cerebellar tumor	Reduced tumor formation	?	Yes	GLI1 mRNA	Change in structure of mRNA of glioma associated oncogene 1 (GLI1)	[73]
Dyschromatosis symmetrica hereditaria (DSH)	Autoimmune	P150	Yes	Endogenous dsRNA	Interferon; Inhibition of MDA5-MAVS	[34]
Epilepsy, acute spinal cord injury	Neurological	?	Yes	GRIA2 mRNA	Enhanced synaptic responses	[88,89,90,91]
Epstein-Barr virus	Anti-viral	?	Yes	Viral miR-BART6-5p	Suppression of RNA-induced silencing complex (RISC) loading, inhibition of Dicer	[114]
Epstein-Barr virus	Switch to lytic cycle	?	Yes	OriP transcripts	Inhibition of miRNA processing; direct interaction between OriPtL and ADAR1	[55]
Esophageal squamous cell cancer (ESCC)	Cancer cell proliferation	P110	Yes	FLNB mRNA	ADAR1 gene amplification	[67]
Gastric cancer, Non-squamous cell lung carcinoma (NSCLC), colorectal cancer	Metastasis	?	Yes	RHOQ mRNA	?	[80]
Hepatocellular carcinoma (HCC), ESCC	Cancer cell proliferation	P110	Yes	AZIN1 mRNA	Increase in ADAR1 levels and editing	[63]
Hepatitis C virus	Antiviral	P150	Yes	Viral RNA	Degradation of edited RNA	[54]
Hepatitis D virus	Switch from proliferation to packaging	P110	Yes	Viral HDAg-S gene	Disruption of translation termination	[52,53]
HIV-1	Proviral	P110 and P150	Yes	Viral p24 Gag, Rev, and Tat mRNA	Increase in ADAR1 expression	[57,58,59,60,135]
HIV-1	Pro-viral	P150	No	-	Inhibition of protein kinase R (PKR) phosphorylation	[60]
HIV-1	Antiviral	?	Yes	HIV-1 envelope glycoprotein	Degradation of edited RNA	[56]
Human T-cell leukemia virus 1 and -2 (HTLV-1 and -2)	Pro-viral	P150	No	-	Inhibition of PKR phosphorylation	[57]
Influenza A virus	Antiviral	?	Yes	Viral ssRNA	TLR7 sensing; IFN	[50]
Locomotion, learning disorders	Neurological	?	Yes	CAPS1 mRNA	Increase in catecholamine secretion	[81]
Lung cancer	Metastasis	?	Yes	miR-381	?	[71]
Measles virus	Proviral	P150	Yes	Viral DI-RNA	Destabilization of DI-RNA	[49]
Measles virus	Pro-viral	P150	No	-	Inhibition of PKR phosphorylation	[63]
Measles virus	Anti-viral	P150	Yes	?	?	[50]
Enhanced metabolism Depression	Neurological	P110	Yes	HTR2C mRNA	Synaptic transmission	[82,83]
Metastatic melanoma, gastric, thyroid and lung cancers	Suppress metastasis	?	Yes	Pri-miR-455-5p	miRNA processing	[103]
NSCLC	Cancer cell proliferation	?	Yes	NEIL1 mRNA	ADAR1 gene amplification	[72]
Pancreatic cancer, papillary thyroid carcinoma, prostate carcinoma and metastatic melanoma	Cancer, metastasis	?	No	miR-221/222	Decrease in miR-221/222 synthesis	[119]
Prostate cancer	Malignancy	?	Yes	PCA3/PRUNE2 complex	mRNA translation	[133]
Spastic paraplegia	Autoimmune	?	Yes	Endogenous dsRNA	Interferon; Inhibition of MDA5-MAVS	[37]
Subacute sclerosing panencephalitis (SSPE) in measles virus	Decreased viral assembly and release	?	Yes	Viral M gene	Inhibition of M protein production	[50]
Vesicular stomatitis virus	Pro-viral	P150	No	-	Inhibition of PKR phosphorylation	[22]

* “?” indicates that the information is currently unknown.

## References

[1] Bass B.L., Weintraub H. (1987). A developmentally regulated activity that unwinds RNA duplexes. Cell.

[2] Rebagliati M.R., Melton D.A. (1987). Antisense RNA injections in fertilized frog eggs reveal an RNA duplex unwinding activity. Cell.

[3] Bass B.L., Weintraub H. (1988). An unwinding activity that covalently modifies its double-stranded RNA substrate. Cell.

[4] Wagner R.W., Nishikura K. (1988). Cell cycle expression of RNA duplex unwindase activity in mammalian cells. Mol. Cell. Biol..

[5] Bass B.L. (1997). RNA editing and hypermutation by adenosine deamination. Trends Biochem. Sci..

[6] Polson A.G., Bass B.L. (1994). Preferential selection of adenosines for modification by double-stranded RNA adenosine deaminase. EMBO J..

[7] Lehmann K.A., Bass B.L. (2000). Double-stranded RNA adenosine deaminases ADAR1 and ADAR2 have overlapping specificities. Biochemistry.

[8] Stefl R., Oberstrass F.C., Hood J.L., Jourdan M., Zimmermann M., Skrisovska L., Maris C., Peng L., Hofr C., Emeson R.B. (2010). The solution structure of the ADAR2 dsrbm-RNA complex reveals a sequence-specific readout of the minor groove. Cell.

[9] Barraud P., Allain F.H. (2012). Adar proteins: Double-stranded RNA and z-DNA binding domains. Curr. Top. Microbiol. Immunol..

[10] Kim U., Wang Y., Sanford T., Zeng Y., Nishikura K. (1994). Molecular cloning of cdna for double-stranded RNA adenosine deaminase, a candidate enzyme for nuclear RNA editing. Proc. Natl. Acad. Sci. USA.

[11] Melcher T., Maas S., Herb A., Sprengel R., Seeburg P.H., Higuchi M. (1996). A mammalian RNA editing enzyme. Nature.

[12] Chen C.X., Cho D.S., Wang Q., Lai F., Carter K.C., Nishikura K. (2000). A third member of the RNA-specific adenosine deaminase gene family, ADAR3, contains both single- and double-stranded RNA binding domains. RNA.

[13] Melcher T., Maas S., Herb A., Sprengel R., Higuchi M., Seeburg P.H. (1996). Red2, a brain-specific member of the RNA-specific adenosine deaminase family. J. Biol. Chem..

[14] Poulsen H., Nilsson J., Damgaard C.K., Egebjerg J., Kjems J. (2001). CRM1 mediates the export of ADAR1 through a nuclear export signal within the Z-DNA binding domain. Mol. Cell. Biol.

[15] George C.X., Samuel C.E. (1999). Human RNA-specific adenosine deaminase ADAR1 transcripts possess alternative exon 1 structures that initiate from different promoters, one constitutively active and the other interferon inducible. Proc. Natl. Acad. Sci. USA.

[16] Strehblow A., Hallegger M., Jantsch M.F. (2002). Nucleocytoplasmic distribution of human RNA-editing enzyme ADAR1 is modulated by double-stranded RNA-binding domains, a leucine-rich export signal, and a putative dimerization domain. Mol. Biol. Cell.

[17] Desterro J.M., Keegan L.P., Lafarga M., Berciano M.T., O′Connell M., Carmo-Fonseca M. (2003). Dynamic association of RNA-editing enzymes with the nucleolus. J. Cell Sci..

[18] Nishikura K. (2016). A-to-I editing of coding and non-coding RNAs by ADARS. Nat. Rev. Mol. Cell Biol..

[19] Nishikura K. (2010). Functions and regulation of RNA editing by ADAR deaminases. Annu. Rev. Biochem..

[20] Wang Q., Khillan J., Gadue P., Nishikura K. (2000). Requirement of the RNA editing deaminase ADAR1 gene for embryonic erythropoiesis. Science.

[21] Yang J.H., Luo X., Nie Y., Su Y., Zhao Q., Kabir K., Zhang D., Rabinovici R. (2003). Widespread inosine-containing mRNA in lymphocytes regulated by ADAR1 in response to inflammation. Immunology.

[22] Liddicoat B.J., Chalk A.M., Walkley C.R. (2016). ADAR1, inosine and the immune sensing system: Distinguishing self from non-self. Wiley Interdiscip. Rev. RNA.

[23] Yang S., Deng P., Zhu Z., Zhu J., Wang G., Zhang L., Chen A.F., Wang T., Sarkar S.N., Billiar T.R. (2014). Adenosine deaminase acting on RNA 1 limits RIG-I RNA detection and suppresses IFN production responding to viral and endogenous RNAs. J. Immunol..

[24] Pestal K., Funk C.C., Snyder J.M., Price N.D., Treuting P.M., Stetson D.B. (2015). Isoforms of RNA-editing enzyme ADAR1 independently control nucleic acid sensor MDA5-driven autoimmunity and multi-organ development. Immunity.

[25] Liddicoat B.J., Piskol R., Chalk A.M., Ramaswami G., Higuchi M., Hartner J.C., Li J.B., Seeburg P.H., Walkley C.R. (2015). RNA editing by ADAR1 prevents MDA5 sensing of endogenous dsRNA as nonself. Science.

[26] George C.X., Ramaswami G., Li J.B., Samuel C.E. (2016). Editing of cellular self-RNAs by adenosine deaminase ADAR1 suppresses innate immune stress responses. J. Biol. Chem..

[27] Patterson J.B., Samuel C.E. (1995). Expression and regulation by interferon of a double-stranded-RNA-specific adenosine deaminase from human cells: Evidence for two forms of the deaminase. Mol. Cell. Biol..

[28] Hartner J.C., Schmittwolf C., Kispert A., Muller A.M., Higuchi M., Seeburg P.H. (2004). Liver disintegration in the mouse embryo caused by deficiency in the RNA-editing enzyme ADAR1. J. Biol. Chem..

[29] Hartner J.C., Walkley C.R., Lu J., Orkin S.H. (2009). ADAR1 is essential for the maintenance of hematopoiesis and suppression of interferon signaling. Nat. Immunol..

[30] XuFeng R., Boyer M.J., Shen H., Li Y., Yu H., Gao Y., Yang Q., Wang Q., Cheng T. (2009). ADAR1 is required for hematopoietic progenitor cell survival via RNA editing. Proc. Natl. Acad. Sci. USA.

[31] Mannion N.M., Greenwood S.M., Young R., Cox S., Brindle J., Read D., Nellaker C., Vesely C., Ponting C.P., McLaughlin P.J. (2014). The RNA-editing enzyme ADAR1 controls innate immune responses to RNA. Cell Rep..

[32] Crow Y.J., Chase D.S., Lowenstein Schmidt J., Szynkiewicz M., Forte G.M., Gornall H.L., Oojageer A., Anderson B., Pizzino A., Helman G. (2015). Characterization of human disease phenotypes associated with mutations in TREX1, RNASEH2A, RNASEH2B, RNASEH2C, SAMHD1, ADAR, and IFIH1. Am. J. Med. Genet. A.

[33] Lebon P., Badoual J., Ponsot G., Goutieres F., Hemeury-Cukier F., Aicardi J. (1988). Intrathecal synthesis of interferon-alpha in infants with progressive familial encephalopathy. J. Neurol. Sci..

[34] Li M., Yang L., Li C., Jin C., Lai M., Zhang G., Hu Y., Ji J., Yao Z. (2010). Mutational spectrum of the ADAR1 gene in dyschromatosis symmetrica hereditaria. Arch. Dermatol. Res..

[35] Kondo T., Suzuki T., Mitsuhashi Y., Ito S., Kono M., Komine M., Akita H., Tomita Y. (2008). Six novel mutations of the ADAR1 gene in patients with dyschromatosis symmetrica hereditaria: Histological observation and comparison of genotypes and clinical phenotypes. J. Dermatol..

[36] Livingston J.H., Lin J.P., Dale R.C., Gill D., Brogan P., Munnich A., Kurian M.A., Gonzalez-Martinez V., De Goede C.G., Falconer A. (2014). A type I interferon signature identifies bilateral striatal necrosis due to mutations in adar1. J. Med. Genet..

[37] Crow Y.J., Zaki M.S., Abdel-Hamid M.S., Abdel-Salam G., Boespflug-Tanguy O., Cordeiro N.J., Gleeson J.G., Gowrinathan N.R., Laugel V., Renaldo F. (2014). Mutations in ADAR1, IFIH1, and RNASEH2B presenting as spastic paraplegia. Neuropediatrics.

[38] Rice G.I., Kasher P.R., Forte G.M., Mannion N.M., Greenwood S.M., Szynkiewicz M., Dickerson J.E., Bhaskar S.S., Zampini M., Briggs T.A. (2012). Mutations in ADAR1 cause aicardi-goutieres syndrome associated with a type I interferon signature. Nat. Genet..

[39] Hayashi M., Suzuki T. (2013). Dyschromatosis symmetrica hereditaria. J. Dermatol..

[40] Zhang S., Jiang M., Zhao J. (2013). Two novel mutations in the DSRAD gene in two chinese pedigrees with dyschromatosis symmetrica hereditaria. Eur. J. Dermatol..

[41] Zhu C.Y., Zhu K.J., Zhou Y., Fan Y.M. (2013). A novel insertion mutation in the ADAR1 gene of a Chinese family with dyschromatosis symmetrica hereditaria. Genet. Mol. Res..

[42] Liu Q., Wang Z., Wu Y., Cao L., Tang Q., Xing X., Ma H., Zhang S., Luo Y. (2014). Five novel mutations in the ADAR1 gene associated with dyschromatosis symmetrica hereditaria. BMC Med. Genet..

[43] Kono M., Matsumoto F., Suzuki Y., Suganuma M., Saitsu H., Ito Y., Fujiwara S., Moriwaki S., Matsumoto K., Matsumoto N. (2016). Dyschromatosis symmetrica hereditaria and aicardi-goutieres syndrome 6 are phenotypic variants caused by ADAR1 mutations. J. Invest. Dermatol..

[44] Liu Y., Zhang Z., Mu Y., Xiong F., Chen X., Yang H., Yang P., Liu L. (2016). Two novel mutations of the ADAR1 gene associated with dyschromatosis symmetrica hereditaria. Zhonghua Yi Xue Yi Chuan Xue Za Zhi.

[45] Zhang G., Shao M., Li Z., Gu Y., Du X., Wang X., Li M. (2016). Genetic spectrum of dyschromatosis symmetrica hereditaria in Chinese patients including a novel nonstop mutation in ADAR1 gene. BMC Med. Genet..

[46] Cho D.S., Yang W., Lee J.T., Shiekhattar R., Murray J.M., Nishikura K. (2003). Requirement of dimerization for RNA editing activity of adenosine deaminases acting on RNA. J. Biol. Chem..

[47] Valente L., Nishikura K. (2007). RNA binding-independent dimerization of adenosine deaminases acting on RNA and dominant negative effects of nonfunctional subunits on dimer functions. J. Biol. Chem..

[48] Vitali P., Scadden A.D. (2010). Double-stranded RNAs containing multiple IU pairs are sufficient to suppress interferon induction and apoptosis. Nat. Struct. Mol. Biol..

[49] Pfaller C.K., Mastorakos G.M., Matchett W.E., Ma X., Samuel C.E., Cattaneo R. (2015). Measles virus defective interfering RNAs are generated frequently and early in the absence of C protein and can be destabilized by adenosine deaminase acting on RNA-1-like hypermutations. J. Virol..

[50] Ward S.V., George C.X., Welch M.J., Liou L.Y., Hahm B., Lewicki H., de la Torre J.C., Samuel C.E., Oldstone M.B. (2011). RNA editing enzyme adenosine deaminase is a restriction factor for controlling measles virus replication that also is required for embryogenesis. Proc. Natl. Acad. Sci. USA.

[51] Sarvestani S.T., Tate M.D., Moffat J.M., Jacobi A.M., Behlke M.A., Miller A.R., Beckham S.A., McCoy C.E., Chen W., Mintern J.D. (2014). Inosine-mediated modulation of rna sensing by toll-like receptor 7 (TLR7) and TLR8. J. Virol..

[52] Macnaughton T.B., Li Y.I., Doughty A.L., Lai M.M. (2003). Hepatitis delta virus RNA encoding the large delta antigen cannot sustain replication due to rapid accumulation of mutations associated with RNA editing. J. Virol..

[53] Casey J.L. (2012). Control of ADAR1 editing of hepatitis delta virus RNAs. Curr. Top. Microbiol. Immunol..

[54] Taylor D.R., Puig M., Darnell M.E., Mihalik K., Feinstone S.M. (2005). New antiviral pathway that mediates hepatitis C virus replicon interferon sensitivity through ADAR1. J. Virol..

[55] Cao S., Moss W., O’Grady T., Concha M., Strong M.J., Wang X., Yu Y., Baddoo M., Zhang K., Fewell C. (2015). New noncoding lytic transcripts derived from the epstein-barr virus latency origin of replication, orip, are hyperedited, bind the paraspeckle protein, NONO/p54nrb, and support viral lytic transcription. J. Virol..

[56] Weiden M.D., Hoshino S., Levy D.N., Li Y., Kumar R., Burke S.A., Dawson R., Hioe C.E., Borkowsky W., Rom W.N. (2014). Adenosine deaminase acting on RNA-1 (ADAR1) inhibits HIV-1 replication in human alveolar macrophages. PLoS ONE.

[57] Cachat A., Alais S., Chevalier S.A., Journo C., Fusil F., Dutartre H., Boniface A., Ko N.L., Gessain A., Cosset F.L. (2014). ADAR1 enhances HTLV-1 and HTLV-2 replication through inhibition of PKR activity. Retrovirology.

[58] Cuadrado E., Booiman T., van Hamme J.L., Jansen M.H., van Dort K.A., Vanderver A., Rice G.I., Crow Y.J., Kootstra N.A., Kuijpers T.W. (2015). ADAR1 facilitates HIV-1 replication in primary CD4+ T cells. PLoS ONE.

[59] Phuphuakrat A., Kraiwong R., Boonarkart C., Lauhakirti D., Lee T.H., Auewarakul P. (2008). Double-stranded RNA adenosine deaminases enhance expression of human immunodeficiency virus type 1 proteins. J. Virol..

[60] Doria M., Neri F., Gallo A., Farace M.G., Michienzi A. (2009). Editing of HIV-1 RNA by the double-stranded RNA deaminase ADAR1 stimulates viral infection. Nucleic Acids Res..

[61] Orecchini E., Federico M., Doria M., Arenaccio C., Giuliani E., Ciafre S.A., Michienzi A. (2015). The ADAR1 editing enzyme is encapsidated into HIV-1 virions. Virology.

[62] Munir M., Berg M. (2013). The multiple faces of proteinkinase R in antiviral defense. Virulence.

[63] George C.X., John L., Samuel C.E. (2014). An RNA editor, adenosine deaminase acting on double-stranded RNA (ADAR1). J. Interferon Cytokine Res..

[64] John L., Samuel C.E. (2014). Induction of stress granules by interferon and down-regulation by the cellular RNA adenosine deaminase ADAR1. Virology.

[65] Nie Y., Ding L., Kao P.N., Braun R., Yang J.H. (2005). ADAR1 interacts with NF90 through double-stranded RNA and regulates NF90-mediated gene expression independently of Rna editing. Mol. Cell Biol..

[66] Reichman T.W., Muniz L.C., Mathews M.B. (2002). The RNA binding protein nuclear factor 90 functions as both a positive and negative regulator of gene expression in mammalian cells. Mol. Cell Biol..

[67] Qin Y.R., Qiao J.J., Chan T.H., Zhu Y.H., Li F.F., Liu H., Fei J., Li Y., Guan X.Y., Chen L. (2014). Adenosine-to-inosine RNA editing mediated by ADARs in esophageal squamous cell carcinoma. Cancer Res..

[68] Chan T.H., Lin C.H., Qi L., Fei J., Li Y., Yong K.J., Liu M., Song Y., Chow R.K., Ng V.H. (2014). A disrupted RNA editing balance mediated by ADARs (adenosine deaminases that act on RNA) in human hepatocellular carcinoma. Gut.

[69] Han L., Diao L., Yu S., Xu X., Li J., Zhang R., Yang Y., Werner H.M., Eterovic A.K., Yuan Y. (2015). The genomic landscape and clinical relevance of A-to-I RNA editing in human cancers. Cancer Cell.

[70] Wang Y., Zeng Y., Murray J.M., Nishikura K. (1995). Genomic organization and chromosomal location of the human dsRNA adenosine deaminase gene: The enzyme for glutamate-activated ion channel RNA editing. J. Mol. Biol..

[71] Anadon C., Guil S., Simo-Riudalbas L., Moutinho C., Setien F., Martinez-Cardus A., Moran S., Villanueva A., Calaf M., Vidal A. (2015). Gene amplification-associated overexpression of the RNA editing enzyme ADAR1 enhances human lung tumorigenesis. Oncogene.

[72] Yeo J., Goodman R.A., Schirle N.T., David S.S., Beal P.A. (2010). RNA editing changes the lesion specificity for the DNA repair enzyme neil1. Proc. Natl. Acad. Sci. USA.

[73] Shimokawa T., Rahman M.F., Tostar U., Sonkoly E., Stahle M., Pivarcsi A., Palaniswamy R., Zaphiropoulos P.G. (2013). RNA editing of the GLI1 transcription factor modulates the output of hedgehog signaling. RNA Biol..

[74] Zipeto M.A., Jiang Q., Melese E., Jamieson C.H. (2015). RNA rewriting, recoding, and rewiring in human disease. Trends Mol. Med..

[75] Quelen C., Eloit Y., Noirot C., Bousquet M., Brousset P. (2016). RNA editing in acute myeloid leukaemia with normal karyotype. Br. J. Haematol..

[76] Beghini A., Ripamonti C.B., Peterlongo P., Roversi G., Cairoli R., Morra E., Larizza L. (2000). RNA hyperediting and alternative splicing of hematopoietic cell phosphatase (PTPN6) gene in acute myeloid leukemia. Hum. Mol. Genet..

[77] Gumireddy K., Li A., Kossenkov A.V., Sakurai M., Yan J., Li Y., Xu H., Wang J., Zhang P.J., Zhang L. (2016). The mRNA-edited form of GABRA3 suppresses GABRA3-mediated AKT activation and breast cancer metastasis. Nat. Commun..

[78] Ohlson J., Pedersen J.S., Haussler D., Ohman M. (2007). Editing modifies the GABA(A) receptor subunit α3. RNA.

[79] Daniel C., Veno M.T., Ekdahl Y., Kjems J., Ohman M. (2012). A distant cis acting intronic element induces site-selective RNA editing. Nucleic Acids Res..

[80] Han S.W., Kim H.P., Shin J.Y., Jeong E.G., Lee W.C., Kim K.Y., Park S.Y., Lee D.W., Won J.K., Jeong S.Y. (2014). RNA editing in RHOQ promotes invasion potential in colorectal cancer. J. Exp. Med..

[81] Miyake K., Ohta T., Nakayama H., Doe N., Terao Y., Oiki E., Nagatomo I., Yamashita Y., Abe T., Nishikura K. (2016). CAPS1 RNA editing promotes dense core vesicle exocytosis. Cell Rep..

[82] Kawahara Y., Grimberg A., Teegarden S., Mombereau C., Liu S., Bale T.L., Blendy J.A., Nishikura K. (2008). Dysregulated editing of serotonin 2C receptor mRNAs results in energy dissipation and loss of fat mass. J. Neurosci..

[83] Niswender C.M., Herrick-Davis K., Dilley G.E., Meltzer H.Y., Overholser J.C., Stockmeier C.A., Emeson R.B., Sanders-Bush E. (2001). RNA editing of the human serotonin 5-HT2C receptor. Alterations in suicide and implications for serotonergic pharmacotherapy. Neuropsychopharmacology.

[84] Di Narzo A.F., Kozlenkov A., Roussos P., Hao K., Hurd Y., Lewis D.A., Sibille E., Siever L.J., Koonin E., Dracheva S. (2014). A unique gene expression signature associated with serotonin 2C receptor RNA editing in the prefrontal cortex and altered in suicide. Hum. Mol. Genet..

[85] Anastasio N.C., Stutz S.J., Fox R.G., Sears R.M., Emeson R.B., DiLeone R.J., O′Neil R.T., Fink L.H., Li D., Green T.A. (2014). Functional status of the serotonin 5-HT2C receptor (5-HT2CR) drives interlocked phenotypes that precipitate relapse-like behaviors in cocaine dependence. Neuropsychopharmacology.

[86] Lomeli H., Mosbacher J., Melcher T., Hoger T., Geiger J.R., Kuner T., Monyer H., Higuchi M., Bach A., Seeburg P.H. (1994). Control of kinetic properties of AMPA receptor channels by nuclear RNA editing. Science.

[87] Krampfl K., Schlesinger F., Zorner A., Kappler M., Dengler R., Bufler J. (2002). Control of kinetic properties of GluR2 flop AMPA-type channels: Impact of R/G nuclear editing. Eur. J. Neurosci..

[88] Vollmar W., Gloger J., Berger E., Kortenbruck G., Kohling R., Speckmann E.J., Musshoff U. (2004). RNA editing (R/G site) and flip-flop splicing of the AMPA receptor subunit GluR2 in nervous tissue of epilepsy patients. Neurobiol. Dis..

[89] Barbon A., Fumagalli F., Caracciolo L., Madaschi L., Lesma E., Mora C., Carelli S., Slotkin T.A., Racagni G., Di Giulio A.M. (2010). Acute spinal cord injury persistently reduces R/G RNA editing of AMPA receptors. J. Neurochem..

[90] Penn A.C., Balik A., Greger I.H. (2013). Steric antisense inhibition of AMPA receptor Q/R editing reveals tight coupling to intronic editing sites and splicing. Nucleic Acids Res..

[91] Bonini D., Filippini A., La Via L., Fiorentini C., Fumagalli F., Colombi M., Barbon A. (2015). Chronic glutamate treatment selectively modulates AMPA RNA editing and ADAR expression and activity in primary cortical neurons. RNA Biol..

[92] Mahajan S.S., Thai K.H., Chen K., Ziff E. (2011). Exposure of neurons to excitotoxic levels of glutamate induces cleavage of the RNA editing enzyme, adenosine deaminase acting on RNA 2, and loss of GluR2 editing. Neuroscience.

[93] Sailer A., Swanson G.T., Perez-Otano I., O’Leary L., Malkmus S.A., Dyck R.H., Dickinson-Anson H., Schiffer H.H., Maron C., Yaksh T.L. (1999). Generation and analysis of GluR5(Q636R) kainate receptor mutant mice. J. Neurosci..

[94] Egebjerg J., Heinemann S.F. (1993). Ca2+ permeability of unedited and edited versions of the kainate selective glutamate receptor GluR6. Proc. Natl. Acad. Sci. USA.

[95] Kohler M., Burnashev N., Sakmann B., Seeburg P.H. (1993). Determinants of Ca2+ permeability in both TM1 and TM2 of high affinity kainate receptor channels: Diversity by RNA editing. Neuron.

[96] Riedmann E.M., Schopoff S., Hartner J.C., Jantsch M.F. (2008). Specificity of ADAR-mediated RNA editing in newly identified targets. RNA.

[97] Matthews M.M., Thomas J.M., Zheng Y., Tran K., Phelps K.J., Scott A.I., Havel J., Fisher A.J., Beal P.A. (2016). Structures of human ADAR2 bound to dsRNA reveal base-flipping mechanism and basis for site selectivity. Nat. Struct. Mol. Biol..

[98] Wong S.K., Sato S., Lazinski D.W. (2001). Substrate recognition by ADAR1 and ADAR2. RNA.

[99] Nishikura K., Yoo C., Kim U., Murray J.M., Estes P.A., Cash F.E., Liebhaber S.A. (1991). Substrate specificity of the dsRNA unwinding/modifying activity. EMBO J..

[100] Macbeth M.R., Lingam A.T., Bass B.L. (2004). Evidence for auto-inhibition by the N terminus of hADAR2 and activation by dsRNA binding. RNA.

[101] Ha M., Kim V.N. (2014). Regulation of microRNA biogenesis. Nat. Rev. Mol. Cell Biol..

[102] Nemlich Y., Greenberg E., Ortenberg R., Besser M.J., Barshack I., Jacob-Hirsch J., Jacoby E., Eyal E., Rivkin L., Prieto V.G. (2013). Microrna-mediated loss of ADAR1 in metastatic melanoma promotes tumor growth. J. Clin. Invest..

[103] Zhang L., Yang C.S., Varelas X., Monti S. (2016). Altered RNA editing in 3′ UTR perturbs microRNA-mediated regulation of oncogenes and tumor-suppressors. Sci. Rep..

[104] Shoshan E., Mobley A.K., Braeuer R.R., Kamiya T., Huang L., Vasquez M.E., Salameh A., Lee H.J., Kim S.J., Ivan C. (2015). Reduced adenosine-to-inosine miR-455–5p editing promotes melanoma growth and metastasis. Nat. Cell Biol..

[105] Li N.F., Gemenetzidis E., Marshall F.J., Davies D., Yu Y., Frese K., Froeling F.E., Woolf A.K., Feakins R.M., Naito Y. (2013). RhoC interacts with integrin α5β1 and enhances its trafficking in migrating pancreatic carcinoma cells. PLoS ONE.

[106] Islam M., Sharma S., Teknos T.N. (2014). RhoC regulates cancer stem cells in head and neck squamous cell carcinoma by overexpressing IL-6 and phosphorylation of STAT3. PLoS ONE.

[107] Rosenthal D.T., Zhang J., Bao L., Zhu L., Wu Z., Toy K., Kleer C.G., Merajver S.D. (2012). Rhoc impacts the metastatic potential and abundance of breast cancer stem cells. PLoS ONE.

[108] Gembarska A., Luciani F., Fedele C., Russell E.A., Dewaele M., Villar S., Zwolinska A., Haupt S., de Lange J., Yip D. (2012). MDM4 is a key therapeutic target in cutaneous melanoma. Nat. Med..

[109] McEvoy J., Ulyanov A., Brennan R., Wu G., Pounds S., Zhang J., Dyer M.A. (2012). Analysis of MDM2 and MDM4 single nucleotide polymorphisms, mRNA splicing and protein expression in retinoblastoma. PLoS ONE.

[110] Liang H.Q., Wang R.J., Diao C.F., Li J.W., Su J.L., Zhang S. (2015). The PTTG1-targeting miRNAs mir-329, miR-300, miR-381, and miR-655 inhibit pituitary tumor cell tumorigenesis and are involved in a p53/PTTG1 regulation feedback loop. Oncotarget.

[111] Formosa A., Markert E.K., Lena A.M., Italiano D., Finazzi-Agro E., Levine A.J., Bernardini S., Garabadgiu A.V., Melino G., Candi E. (2014). MicroRNAs, mir-154, mir-299–5p, mir-376a, mir-376c, mir-377, mir-381, mir-487b, mir-485–3p, mir-495 and mir-654–3p, mapped to the 14q32.31 locus, regulate proliferation, apoptosis, migration and invasion in metastatic prostate cancer cells. Oncogene.

[112] Garcia-Lopez J., Hourcade Jde D., Del Mazo J. (2013). Reprogramming of microRNAs by adenosine-to-inosine editing and the selective elimination of edited microRNA precursors in mouse oocytes and preimplantation embryos. Nucleic Acids Res..

[113] Kawahara Y., Zinshteyn B., Chendrimada T.P., Shiekhattar R., Nishikura K. (2007). RNA editing of the microRNA-151 precursor blocks cleavage by the Dicer-TRBP complex. EMBO Rep..

[114] Iizasa H., Wulff B.E., Alla N.R., Maragkakis M., Megraw M., Hatzigeorgiou A., Iwakiri D., Takada K., Wiedmer A., Showe L. (2010). Editing of Epstein-Barr virus-encoded BART6 micrornas controls their dicer targeting and consequently affects viral latency. J. Biol. Chem..

[115] Kawahara Y., Zinshteyn B., Sethupathy P., Iizasa H., Hatzigeorgiou A.G., Nishikura K. (2007). Redirection of silencing targets by adenosine-to-inosine editing of mirnas. Science.

[116] Heale B.S., Keegan L.P., McGurk L., Michlewski G., Brindle J., Stanton C.M., Caceres J.F., O′Connell M.A. (2009). Editing independent effects of ADARs on the miRNA/siRNA pathways. EMBO J..

[117] Bahn J.H., Ahn J., Lin X., Zhang Q., Lee J.H., Civelek M., Xiao X. (2015). Genomic analysis of ADAR1 binding and its involvement in multiple RNA processing pathways. Nat. Commun..

[118] Chen T., Xiang J.F., Zhu S., Chen S., Yin Q.F., Zhang X.O., Zhang J., Feng H., Dong R., Li X.J. (2015). Adar1 is required for differentiation and neural induction by regulating microRNA processing in a catalytically independent manner. Cell Res..

[119] Galore-Haskel G., Nemlich Y., Greenberg E., Ashkenazi S., Hakim M., Itzhaki O., Shoshani N., Shapira-Fromer R., Ben-Ami E., Ofek E. (2015). A novel immune resistance mechanism of melanoma cells controlled by the ADAR1 enzyme. Oncotarget.

[120] Ota H., Sakurai M., Gupta R., Valente L., Wulff B.E., Ariyoshi K., Iizasa H., Davuluri R.V., Nishikura K. (2013). ADAR1 forms a complex with dicer to promote microRNA processing and RNA-induced gene silencing. Cell.

[121] Ramaswami G., Zhang R., Piskol R., Keegan L.P., Deng P., O′Connell M.A., Li J.B. (2013). Identifying RNA editing sites using RNA sequencing data alone. Nat. Methods.

[122] Bazak L., Haviv A., Barak M., Jacob-Hirsch J., Deng P., Zhang R., Isaacs F.J., Rechavi G., Li J.B., Eisenberg E. (2014). A-to-I RNA editing occurs at over a hundred million genomic sites, located in a majority of human genes. Genome Res..

[123] Lev-Maor G., Sorek R., Levanon E.Y., Paz N., Eisenberg E., Ast G. (2007). RNA-editing-mediated exon evolution. Genome Biol..

[124] Sakurai M., Yano T., Kawabata H., Ueda H., Suzuki T. (2010). Inosine cyanoethylation identifies a-to-i rna editing sites in the human transcriptome. Nat. Chem Biol.

[125] Ivanov A., Memczak S., Wyler E., Torti F., Porath H.T., Orejuela M.R., Piechotta M., Levanon E.Y., Landthaler M., Dieterich C. (2015). Analysis of intron sequences reveals hallmarks of circular RNA biogenesis in animals. Cell Rep..

[126] Hansen T.B., Jensen T.I., Clausen B.H., Bramsen J.B., Finsen B., Damgaard C.K., Kjems J. (2013). Natural RNA circles function as efficient microRNA sponges. Nature.

[127] Hansen T.B., Kjems J., Damgaard C.K. (2013). Circular RNA and mir-7 in cancer. Cancer Res..

[128] Liang H., Landweber L.F. (2007). Hypothesis: RNA editing of microRNA target sites in humans?. RNA.

[129] Borchert G.M., Gilmore B.L., Spengler R.M., Xing Y., Lanier W., Bhattacharya D., Davidson B.L. (2009). Adenosine deamination in human transcripts generates novel microrna binding sites. Hum. Mol. Genet..

[130] Stellos K., Gatsiou A., Stamatelopoulos K., Perisic Matic L., John D., Lunella F.F., Jae N., Rossbach O., Amrhein C., Sigala F. (2016). Adenosine-to-inosine RNA editing controls cathepsin S expression in atherosclerosis by enabling hur-mediated post-transcriptional regulation. Nat. Med..

[131] Daniel C., Silberberg G., Behm M., Ohman M. (2014). Alu elements shape the primate transcriptome by cis-regulation of RNA editing. Genome Biol.

[132] Osenberg S., Paz Yaacov N., Safran M., Moshkovitz S., Shtrichman R., Sherf O., Jacob-Hirsch J., Keshet G., Amariglio N., Itskovitz-Eldor J. (2010). Alu sequences in undifferentiated human embryonic stem cells display high levels of A-to-I RNA editing. PLoS ONE.

[133] Rinn J.L., Chang H.Y. (2012). Genome regulation by long noncoding RNAs. Annu. Rev. Biochem..

[134] Salameh A., Lee A.K., Cardo-Vila M., Nunes D.N., Efstathiou E., Staquicini F.I., Dobroff A.S., Marchio S., Navone N.M., Hosoya H. (2015). PRUNE2 is a human prostate cancer suppressor regulated by the intronic long noncoding RNA PCA3. Proc. Natl. Acad. Sci. USA.

[135] Orecchini E., Doria M., Antonioni A., Galardi S., Ciafre S.A., Frassinelli L., Mancone C., Montaldo C., Tripodi M., Michienzi A. (2016). ADAR1 restricts LINE-1 retrotransposition. Nucleic Acids Res..

[136] Miki Y., Nishisho I., Horii A., Miyoshi Y., Utsunomiya J., Kinzler K.W., Vogelstein B., Nakamura Y. (1992). Disruption of the APC gene by a retrotransposal insertion of L1 sequence in a colon cancer. Cancer Res..

[137] Iskow R.C., McCabe M.T., Mills R.E., Torene S., Pittard W.S., Neuwald A.F., Van Meir E.G., Vertino P.M., Devine S.E. (2010). Natural mutagenesis of human genomes by endogenous retrotransposons. Cell.

[138] Beck C.R., Garcia-Perez J.L., Badge R.M., Moran J.V. (2011). Line-1 elements in structural variation and disease. Annu. Rev. Genomics Hum. Genet..

[139] Paz-Yaacov N., Bazak L., Buchumenski I., Porath H.T., Danan-Gotthold M., Knisbacher B.A., Eisenberg E., Levanon E.Y. (2015). Elevated RNA editing activity is a major contributor to transcriptomic diversity in tumors. Cell Rep..

[140] Cenci C., Barzotti R., Galeano F., Corbelli S., Rota R., Massimi L., Di Rocco C., O′Connell M.A., Gallo A. (2008). Down-regulation of RNA editing in pediatric astrocytomas: ADAR2 editing activity inhibits cell migration and proliferation. J. Biol. Chem..

[141] Ma C.H., Chong J.H., Guo Y., Zeng H.M., Liu S.Y., Xu L.L., Wei J., Lin Y.M., Zhu X.F., Zheng G.G. (2011). Abnormal expression of ADAR1 isoforms in Chinese pediatric acute leukemias. Biochem. Biophys. Res. Commun..

[142] Chan T.H., Qamra A., Tan K.T., Guo J., Yang H., Qi L., Lin J.S., Ng V.H., Song Y., Hong H. (2016). Adar-mediated RNA editing predicts progression and prognosis of gastric cancer. Gastroenterology.

[143] Dou N., Yu S., Ye X., Yang D., Li Y., Gao Y. (2016). Aberrant overexpression of ADAR1 promotes gastric cancer progression by activating mTOR/p70S6K signaling. Oncotarget.

[144] Zipeto M.A., Court A.C., Sadarangani A., Delos Santos N.P., Balaian L., Chun H.J., Pineda G., Morris S.R., Mason C.N., Geron I. (2016). ADAR1 activation drives leukemia stem cell self-renewal by impairing Let-7 biogenesis. Cell Stem Cell.

[145] Jiang Q., Crews L.A., Barrett C.L., Chun H.J., Court A.C., Isquith J.M., Zipeto M.A., Goff D.J., Minden M., Sadarangani A. (2013). ADAR1 promotes malignant progenitor reprogramming in chronic myeloid leukemia. Proc. Natl. Acad. Sci. USA.

[146] Yang B., Hu P., Lin X., Han W., Zhu L., Tan X., Ye F., Wang G., Wu F., Yin B. (2015). PTBP1 induces ADAR1 p110 isoform expression through IRES-like dependent translation control and influences cell proliferation in gliomas. Cell Mol. Life Sci..

